# Usefulness of automated assays for detecting hepatitis B and C markers in dried blood spot samples

**DOI:** 10.1186/s13104-019-4547-y

**Published:** 2019-08-20

**Authors:** Livia Melo Villar, Helena Medina Cruz, Raissa Martins Deodato, Juliana Custódio Miguel, Elisangela Ferreira da Silva, Geane Lopes Flores, Lia Laura Lewis-Ximenez

**Affiliations:** Viral Hepatitis Laboratory, Oswaldo Cruz Institute, FIOCRUZ, Hélio and Peggy Pereira Pavilion - Ground Floor - Office B09, FIOCRUZ Av. Brasil, 4365 – Manguinhos, Rio de Janeiro, RJ 210360-040 Brazil

**Keywords:** Hepatitis C virus, Diagnosis, Dried blood spot, ECLIA

## Abstract

**Objective:**

Dried blood spots (DBSs) can be used as an alternative to serum samples because they are easily collected and can be transported without refrigeration to reference laboratories for diagnosis. The present study was performed to evaluate the utility of electrochemiluminescence immunoassay “ECLIA” for anti-HCV, HBsAg and anti-HBc detection from DBS samples.

**Results:**

Anti-HCV was detected in 103 DBS samples from 108 paired, positive serum and undetected in 364 DBS samples from 366 paired, negative specimens, giving a sensitivity of 95.4% and a specificity of 99.4%. HBsAg was detected in 67 DBS samples out of 71 positive, paired serum and was undetected among 295 DBS samples from 298 paired, negative specimens, giving a sensitivity and specificity of 94.4% and 99%, respectively. Anti-HBc was detected in 160 DBS samples from 185 paired, positive serum specimens and undetected in 349 DBS samples from 357 paired, negative serum specimens, giving a sensitivity of 86.5% and a specificity of 97.8%. Overall, the Kappa index indicated a high agreement between results obtained for the serum and DBS samples (k: 0.95, 0.93 and 0.86 for anti-HCV, HBsAg, anti-HBc, respectively). In conclusion, the ECLIA test could be used for detecting hepatitis B and C markers in DBS.

## Introduction

Hepatitis B and C virus infections are responsible for more than 300 million chronically infected individuals worldwide [1, 2]. Brazil is the largest country in Latin America, where more than 500,000 cases of hepatitis B or C were reported to the Brazilian Health Ministry from 1999 to 2017 [3]. However, the real prevalence of these infections is likely higher, as the country occupies an extensive 8.5 million km^2^ area, including many remote regions which suffer from little to no healthcare access.

Hepatitis B and C diagnosis is made by detecting antigens, antibodies or viral genome from serum samples obtained through venous blood collection [4]; however, trained personnel and infrastructure to collect blood are required. Furthermore, low temperature storage conditions and transportation are necessary, which may be difficult to obtain in limited-resource settings. According to WHO guidelines, DBS could be a useful tool to obtain blood samples for diagnosis in vulnerable populations or remote areas [5]. Some studies have reported detection of HBV and HCV markers using manual enzyme immunoassays [6–11], but little is known about the utility of the electrochemiluminescence immunoassay “ECLIA” for anti-HCV, HBsAg and anti-HBc detection from DBS samples. This method is fast, employs a small amount of sample, is highly accurate and can yield results in few minutes.

## Main text

### Methods

This cross-sectional study included 1385 paired plasma and DBS samples (71 HBsAg positive, 185 anti-HBc positive, 108 anti-HCV positive, 298 HBsAg negative, 357 anti-HBc negative, 366 anti-HCV negative) collected at the Viral Hepatitis Clinic (FIOCRUZ, RIO DE JANEIRO, BRAZIL). This study was approved by the Fiocruz Ethics Committee. Informed written consent was obtained from each individual.

The samples were obtained from referred patients and suspected cases received at the clinic to determine hepatitis diagnosis. Paired serum and DBS samples were obtained, where 6 mL whole blood was collected from each patient and 75 µL was spotted on a Whatman 903 protein saver card (Whatman, GE Healthcare, NJ), until 12-mm pre-printed circular paper disks were completely filled. To elute DBS samples, a 12-mm disc of filter paper was cut and transferred to a microtube containing 500 µL of PBS/BSA 0.5% for 18 to 24 h.

After incubation, eluate was directly submitted to the following ECLIA: Elecsys anti-HCV II, Elecsys HBsAg II and Elecsys anti-HBc II (Roche Diagnostics) following the manufacturer’s instructions. In the Anti-HCV and HBsAg assay, samples with sample/cutoff (S/CO) values < 1.0 are considered non-reactive while for anti-HBc assay, non-reactive samples should present S/CO > 1.0.

Descriptive statistics comprise the mean ± the standard deviation, with a preliminary assessment using contingency tables and respective statistics. The results obtained with serum were used as a reference to assess sensitivity, specificity, positive predictive value, negative predictive value and respective confidence interval (CI).

Concordance between the results obtained for the paired DBS and sera samples was assessed using the Kappa index (k). According to international standards, findings should be interpreted as follows: < 0.20 corresponds to poor agreement; 0.21–0.40 fair agreement; 0.41–0.60 moderate agreement; 0.61–0.80 good agreement, and 0.81–1.00 corresponds to very good agreement [12]. Analyses were performed using GraphPad InStat 3.01 (GraphPad Software, San Diego, CA).

### Results

Anti-HCV was detected in 103 DBS samples from 108 paired, positive serum specimens and undetected in 364 DBS samples from 366 paired, negative serum specimens, giving a sensitivity and specificity of 95.4% and 99.4% respectively. HBsAg was detected in 67 DBS samples out of 71 positive, paired serum specimens and was undetected among 295 DBS samples from 298 paired, negative serum specimens, giving a sensitivity and specificity of 94.4% and 99%, respectively. Anti-HBc was detected in 160 DBS samples from 185 paired, positive serum specimens and undetected in 349 DBS samples from 357 paired, negative serum specimens, giving a sensitivity and specificity of 86.5% and 97.8% respectively. Overall, the Kappa index indicated a high agreement between results obtained for the serum and DBS samples (k: 0.95, 0.93 and 0.86 for anti-HCV, HBsAg, anti-HBc, respectively) (Table 1).

**Table 1:** Sensitivity, specificity, negative and positive predictive values and kappa index for anti-HCV, HBsAg and anti-HBs detection in DBS samples using ECLIA

	Anti-HCV		Anti-HBc		HBsAg	
	Value (%)	IC (%)	Value (%)	IC (%)	Value (%)	IC (%)
Sensitivity	95.37	89.53–98.48	86.49	80.70–91.06	94.37	86.20–98.44
Specificity	99.45	98.04–99.93	97.76	95.63–99.03	98.99	97.09–99.79
Positive predictive value	98.10	92.82–99.52	95.24	90.96–97.55	95.71	87.85–98.57
Negative predictive value	98.64	96.87–99.42	93.32	90.65–95.26	98.66	96.61–99.48
Kappa index	0.957	0.926–0.988	0.861	0.815- 0.907	0.938	0.893–0.983

### Discussion

In this study, we evaluated the utility of automated ECLIA for HBsAg, anti-HBc, and anti-HCV detection in DBS samples. High sensitivity and specificity were found for anti-HCV detection comparable to previous study of Marques et al. that used manual EIA for anti-HCV detection (97.5% sensitivity and 99% specificity for RADIM EIA), by McCarron et al. [13] who used EIA Monolisa anti-HCV (100% sensitivity and 87.5% specificity), and Tuaillon et al. [14] who employed an Ortho HCV 3.0 enzyme-linked immunosorbent assay kit on DBS (99% sensitivity and specificity). These results demonstrated the utility of ECLIA in the detection of anti-HCV in DBS as equivalent to that of the enzyme immunoassay, which could increase the access of diagnosis.

Regarding HBsAg detection in DBS using ECLIA, we found high sensitivity (94.37%) and specificity (98.99%) compared to serum results. The same was found by Mossner et al. [15] using the Architect system (Abbott Diagnostics, Delkenheim, Germany) where HBsAg sensitivity was 96.5% and specificity was 99%. Other studies also found sensitivities and specificities above 99% using the chemiluminescent microparticle immunoassay [16–18].

The present study also evaluated anti-HBc marker detection in DBS, which is useful in identifying previous exposure to HBV. In the present study, high specificity (97.76%) was observed and sensitivity of anti-HBc detection was 86.49%, which is similar to the observations of Ross et al. [17] (86.3%) and higher than previously reported by Mossner et al. [15] using the Architect system (Abbott Diagnostics) (68%). The low sensitivity of anti-HBc detection in DBS could be attributable to the presence of other infections, such as HIV. Ross et al. [17] observed high sensitivity of anti-HBc detection when HIV-infected individuals were excluded from the analysis (97.1%). However, data regarding HIV status was not available in the present study.

To our knowledge, this is the first report of the analytical performance characteristics of testing HBsAg, anti-HBc and anti-HCV in DBS eluates with the ROCHE ECLIA system. DBS specimens could be a reliable alternative testing specimen, which may increase hepatitis B and C diagnostic opportunities for rural, remote and hard to reach regions. DBS could be easily collected and transported to reference laboratories for testing using automated assays.

## Limitations

- Lack of clinical data and risk factors of the population studied that could explain false negative or positive results.
- Information regarding HIV or high-risk status, such as intravenous drug use, which could improve analysis of the methodological efficiency.

## Data Availability

The datasets analyzed during the current study are available from the correspondence author upon reasonable request.

## Abbreviations

DBS: dried blood spots
ECLIA: electrochemiluminescence immunoassay
Anti-HCV: antibody against hepatitis C virus
HBsAg: hepatitis B surface antigen
Anti-HBc: antibody against hepatitis B core antigen
S: sample
CO: cut off

## References

[1] World Health Organization. Hepatitis B. https://www.who.int/news-room/fact-sheets/detail/hepatitis-b. Accessed 20 May 2019.

[2] World Health Organization. Hepatitis C. https://www.who.int/news-room/fact-sheets/detail/hepatitis-c. Accessed 20 May 2019.

[3] Ministry of Health—Secretariat of Health Surveillance—Department of Surveillance, Prevention and Control of STIs, HIV/AIDS and Hepatitis Viral (DIAHV). Epidemiological bulletin—viral hepatitis. https://www.aids.gov.br/pt-br/pub/2018/boletim-epidemiologico-de-hepatites-virais-2018. Accessed 20 May 2019.

[4] Villar LM, Cruz HM, Barbosa JR, Bezerra CS, Portilho MM, Scalioni LP (2015). Update on hepatitis B and C virus diagnosis. World J Virol.

[5] World Health Organization. Global health sector strategy on viral hepatitis 2016–2021; 2016. https://www.who.int/hepatitis/strategy2016-2021/ghss-hep/en/. Accessed 20 May 2019.

[6] Cruz HM, Miguel Cruz JC, Da Silva EF, Portilho MM, Marques VA, Lewis-Ximenez LL, Lampe E, Villar LM (2018). Comparison of the performance of enzyme immunoassays for hepatitis B and C detection in dried blood spot. J Immunoassay Immunochem.

[7] Flores GL, Cruz HM, Marques VA, Villela-Nogueira CA, Potsch DV, May SB, Brandão-Mello CE, Pires MMA, Pilotto JH, Pollo-Flores P, Esberard EBC, Ivantes C, Lewis-Ximenez LL, Lampe E, Villar LM (2017). Performance of ANTI-HCV testing in dried blood spots and saliva according to HIV status. J Med Virol.

[8] Lange B, Cohn J, Roberts T, Camp J, Chauffour J, Gummadi N, Ishizaki A, Nagarathnam A, Tuaillon E, van de Perre P, Pichler C, Easterbrook P, Denkinger CM (2017). Diagnostic accuracy of serological diagnosis of hepatitis C and B using dried blood spot samples (DBS): two systematic reviews and meta-analyses. BMC Infect Dis.

[9] Marques BL, Brandão CU, Silva EF, Marques VA, Villela-Nogueira CA, Do Ó KM, de Paula MT, Lewis-Ximenez LL, Lampe E, Villar LM (2012). Dried blood spot samples: optimization of commercial EIAs for hepatitis C antibody detection and stability under different storage conditions. J Med Virol.

[10] Villar LM, de Oliveira JC, Cruz HM, Yoshida CF, Lampe E, Lewis-Ximenez LL (2011). Assessment of dried blood spot samples as a simple method for detection of hepatitis B virus markers. J Med Virol.

[11] Villar LM, Bezerra CS, Cruz HM, Portilho MM, Flores GL (2019). Applicability of oral fluid and dried blood spot for hepatitis B virus diagnosis. Can J Gastroenterol Hepatol.

[12] Altman DG (1991). Practical statistics for medical research.

[13] McCarron B, Fox R, Wilson K, Cameron S, McMenamin J, McGregor G, Pithie A, Golberg D (1999). Hepatitis C antibody detection in dried blood spots. J Viral Hepatitis.

[14] Tuaillon E, Mondain AM, Meroueh F, Ottomani L, Picot MC, Nagot N, Van de Perre P, Ducos J (2010). Dried blood spot for hepatitis C virus serology and molecular testing. Hepatology.

[15] Mössner BK, Staugaard B, Jensen J, Lillevang ST, Christensen PB, Holm DK (2016). Dried blood spots, valid screening for viral hepatitis and human immunodeficiency virus in real-life. World J Gastroenterol.

[16] Mohamed S, Raimondo A, Pénaranda G, Camus C, Ouzan D, Ravet S, Bourlière M, Khiri H, Dukan P, Olive D, Halfon P (2013). Dried blood spot sampling for hepatitis B virus serology and molecular testing. PLoS ONE.

[17] Ross RS, Stambouli O, Gruner N, Marcus U, Cai W, Zhang W (2013). Detection of infections with hepatitis B virus, hepatitis C virus, and human immunodeficiency virus by analyses of dried blood spots—performance characteristics of the ARCHITECT system and two commercial assays for nucleic acid amplification. Virol J.

[18] Kenmoe S, Tagnouokam PAN, Nde CK, Mella-Tamko GF, Njouom R (2018). Using dried blood spot for the detection of HBsAg and anti-HCV antibodies in Cameroon. BMC Res Notes.

